# Supplementary material for: Associations between the C3orf20 rs12496846 Polymorphism and Both Postoperative Analgesia after Orthognathic and Abdominal Surgeries and C3orf20 Gene Expression in the Brain
Source: Pharmaceutics. 2022 Mar 28;14(4):727. doi: 10.3390/pharmaceutics14040727 (PMC9028963; doi:10.3390/pharmaceutics14040727)
Supplement: Supplementary file 1 [file pharmaceutics-14-00727-s001.zip › pharmaceutics-1576525-supplementary.pdf]

pharmaceutics

# Supplementary Materials: Associations between the *C3orf20* rs12496846 polymorphism and both postoperative analgesia after orthognathic and abdominal surgeries and *C3orf20* gene expression in the brain

**Daisuke Nishizawa, Makoto Nagashima, Shinya Kasai, Junko Hasegawa, Kyoko Nakayama, Yuko Ebata, Ken-ichi Fukuda, Tatsuya Ichinohe, Masakazu Hayashida, and Kazutaka Ikeda**

**Table S1.** Demographic and clinical data of the subjects who underwent major abdominal surgery.

| | *n* | Minimum | Maximum | Mean | SD | Median |
|---|---|---|---|---|---|---|
| **Gender** | | | | | | |
| male | 60 | | | | | |
| female | 52 | | | | | |
| **Age** | 112 | 28 | 80 | 63.13 | 10.06 | 63.00 |
| **Height (cm)** | 112 | 133 | 175 | 157.89 | 8.00 | 158.00 |
| **Weight (kg)** | 112 | 38 | 77 | 55.83 | 9.97 | 54.00 |
| **24-h postoperative analgesic use (μg/kg)** | 112 | 0 | 6.48 | 0.77 | 1.15 | 0.00 |
| **Ln-transformed 24-h postoperative analgesic use (μg/kg)** | 112 | 0 | 2.01 | 0.42 | 0.51 | 0.00 |

Data excerpted from Nishizawa et al. (2014) [12].

**Table S2.** Demographic and clinical data of the subjects who underwent painful cosmetic surgery.

| | *n* | Minimum | Maximum | Mean | SD | Median |
|---|---|---|---|---|---|---|
| **Gender** | | | | | | |
| male | 125 | | | | | |
| female | 230 | | | | | |
| **Age** | 355 | 15 | 52 | 25.92 | 7.63 | 23.00 |
| **Height (cm)** | 355 | 143 | 258 | 164.78 | 10.07 | 163.00 |
| **Weight (kg)** | 355 | 38 | 128 | 57.61 | 10.86 | 55.00 |
| **24-h postoperative analgesic use (μg/kg)** | 354 | 0 | 13.82 | 2.93 | 2.57 | 2.27 |
| **Ln-transformed 24-h postoperative analgesic use (μg/kg)** | 354 | 0 | 2.70 | 1.17 | 0.64 | 1.18 |

Data excerpted from Nishizawa et al. (2014) [12].

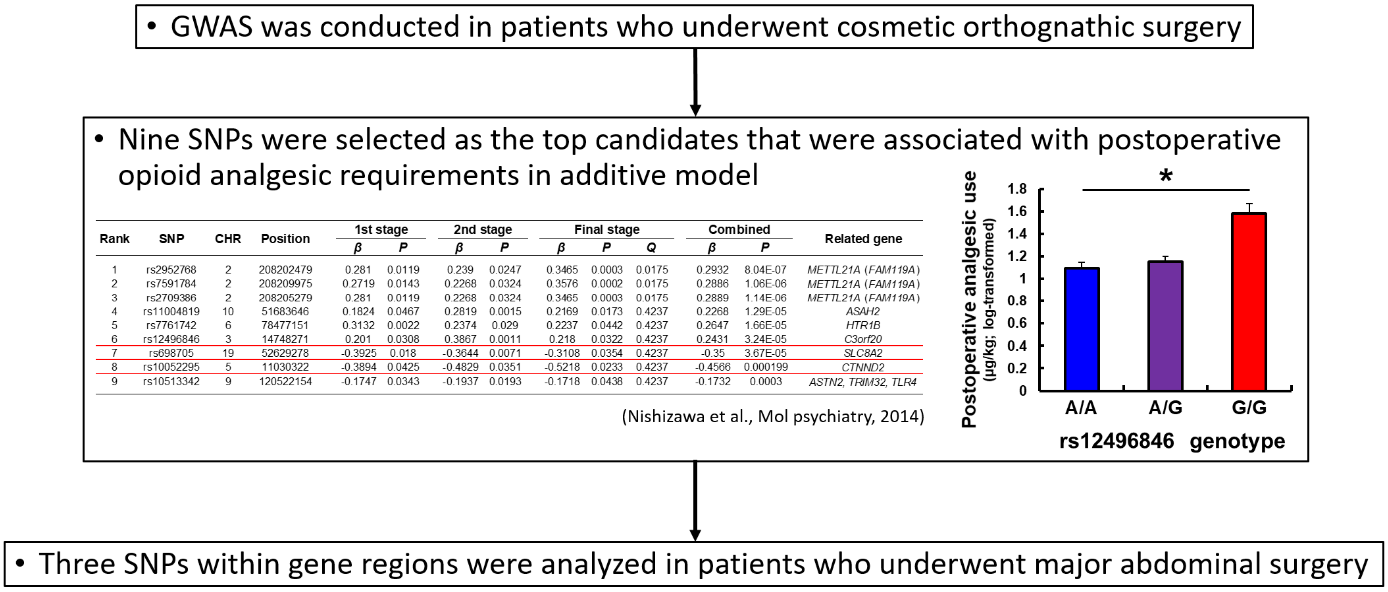


| Rank | SNP | CHR | Position | 1st stage | | 2nd stage | | Final stage | | | Combined | | Related gene |
|---|---|---|---|---|---|---|---|---|---|---|---|---|---|
| | | | | *β* | *P* | *β* | *P* | *β* | *P* | *Q* | *β* | *P* | |
| 1 | rs2952768 | 2 | 208202479 | 0.281 | 0.0119 | 0.239 | 0.0247 | 0.3465 | 0.0003 | 0.0175 | 0.2932 | 8.04E-07 | *METTL21A* (*FAM119A*) |
| 2 | rs7591784 | 2 | 208209975 | 0.2719 | 0.0143 | 0.2268 | 0.0324 | 0.3576 | 0.0002 | 0.0175 | 0.2886 | 1.06E-06 | *METTL21A* (*FAM119A*) |
| 3 | rs2709386 | 2 | 208205279 | 0.281 | 0.0119 | 0.2268 | 0.0324 | 0.3465 | 0.0003 | 0.0175 | 0.2889 | 1.14E-06 | *METTL21A* (*FAM119A*) |
| 4 | rs11004819 | 10 | 51683646 | 0.1824 | 0.0467 | 0.2819 | 0.0015 | 0.2169 | 0.0173 | 0.4237 | 0.2268 | 1.29E-05 | *ASAH2* |
| 5 | rs7761742 | 6 | 78477151 | 0.3132 | 0.0022 | 0.2374 | 0.029 | 0.2237 | 0.0442 | 0.4237 | 0.2647 | 1.66E-05 | *HTR1B* |
| 6 | rs12496846 | 3 | 14748271 | 0.201 | 0.0308 | 0.3867 | 0.0011 | 0.218 | 0.0322 | 0.4237 | 0.2431 | 3.24E-05 | *C3orf20* |
| 7 | rs698705 | 19 | 52629278 | -0.3925 | 0.018 | -0.3644 | 0.0071 | -0.3108 | 0.0354 | 0.4237 | -0.35 | 3.67E-05 | *SLC8A2* |
| 8 | rs10052295 | 5 | 11030322 | -0.3894 | 0.0425 | -0.4829 | 0.0351 | -0.5218 | 0.0233 | 0.4237 | -0.4566 | 0.000199 | *CTNND2* |
| 9 | rs10513342 | 9 | 120522154 | -0.1747 | 0.0343 | -0.1937 | 0.0193 | -0.1718 | 0.0438 | 0.4237 | -0.1732 | 0.0003 | *ASTN2, TRIM32, TLR4* |

**Figure S1.** A graph with scheme of SNPs selection in the present study.

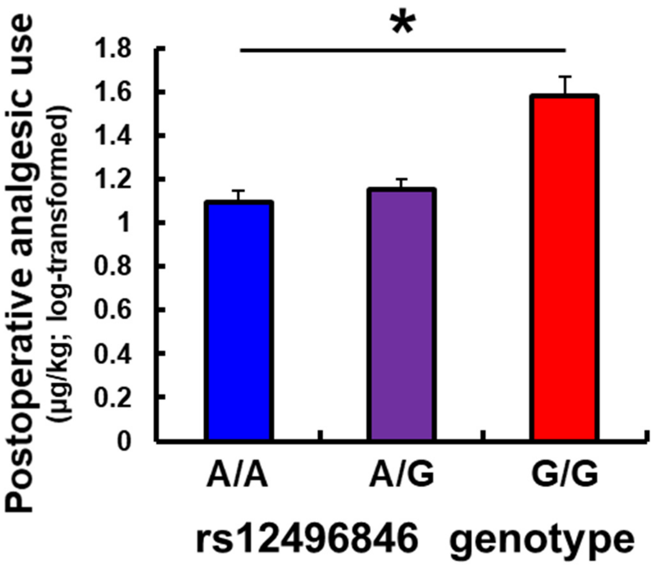


**Figure S2.** Association analysis between postoperative opioid analgesic requirements and the rs12496846 SNP in subjects who underwent painful cosmetic surgery, showing the total dose of fentanyl that was administered per body weight (µg/kg; log transformed) through a patient-controlled analgesia pump during the 24-h postoperative period. *Corrected $p < 0.05$, greater dose of analgesic administered as the number of the G allele of the rs12496846 SNP that was carried in subjects increased. The data are expressed as mean ± SEM.

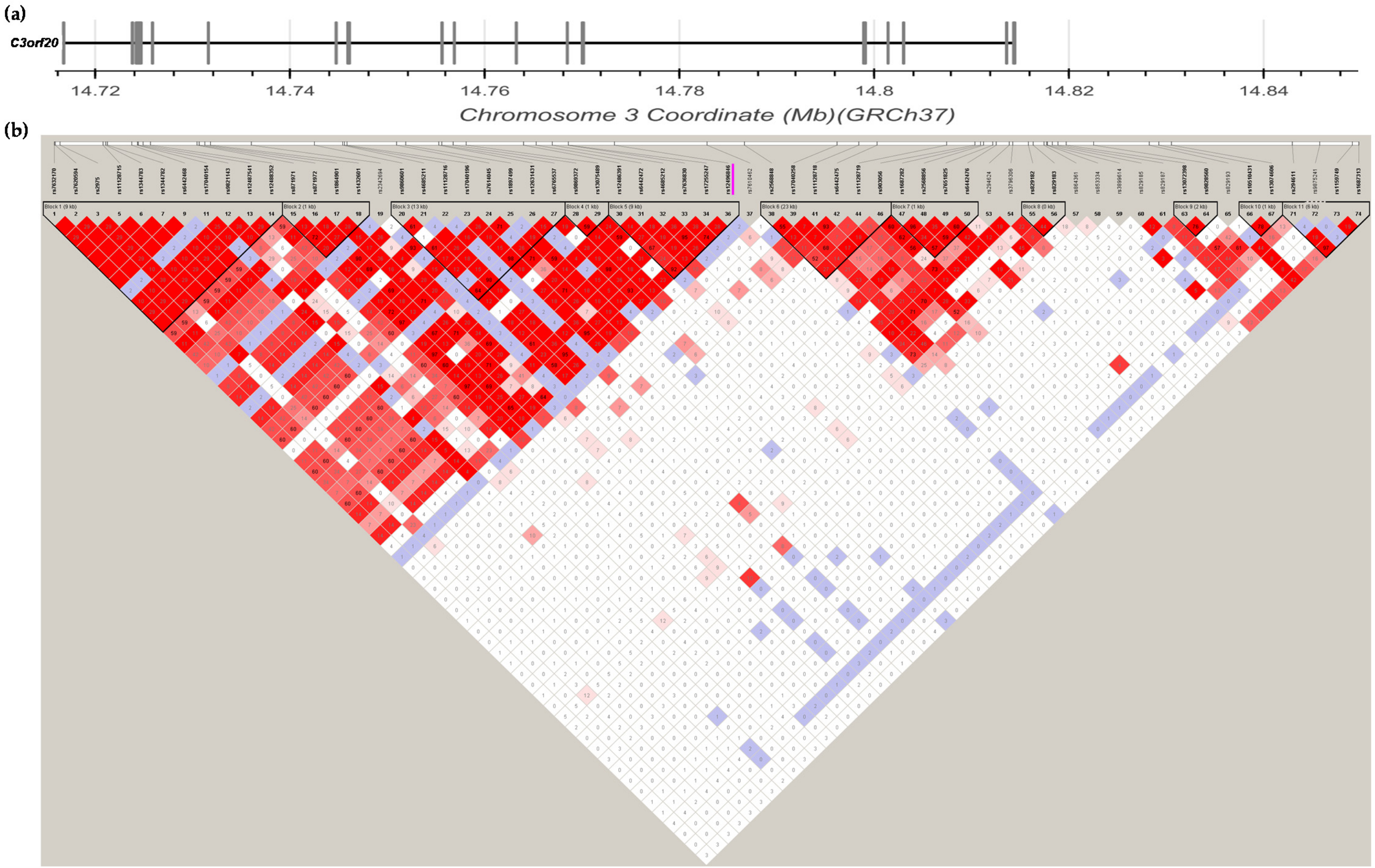


**Figure S3.** Linkage disequilibrium (LD) plot for the *C3orf20* gene and flanking region. (**a**) Illustration of the *C3orf20* gene in the genomic region from position 14715782 to 14849667 on chromosome 3 depicted with the LDmatrix Tool in LDlink (https://ldlink.nci.nih.gov/?tab=home; accessed January 5, 2022). (**b**) State of LD between the SNPs in the genomic position from 14715782 to 14849667 on chromosome 3, based on genotype data of the subjects who were derived from the Japanese population and underwent cosmetic orthognathic surgery. Numbers in squares in which two SNPs face represent the percentage of $r^2$ values that were calculated from genotype data of the SNPs. Red blank squares represent $r^2 = 1$. A pink line indicates the rs12496846 SNP.

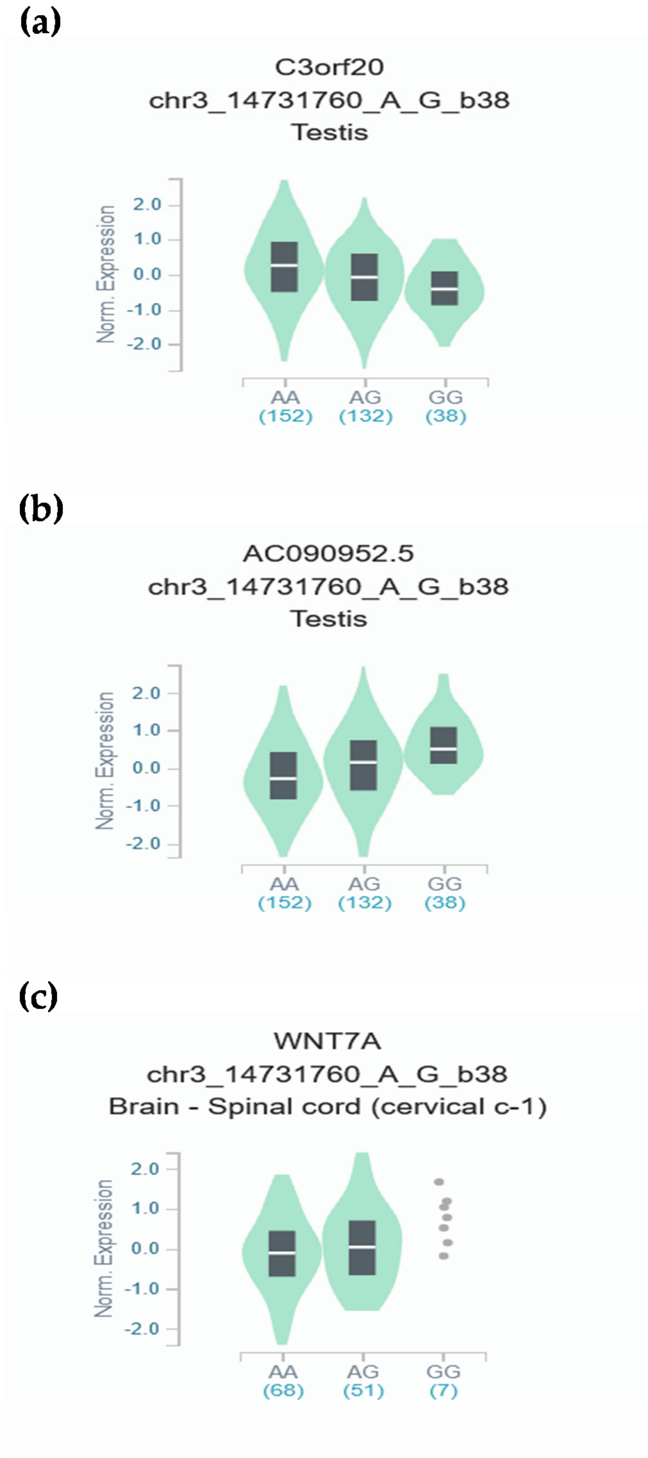


**Figure S4.** Normalized mRNA expression level of genes between each genotype subgroup of the rs12496846 SNP, for which the associations were significant in the GTEx portal database. (**a**) Normalized mRNA expression level of the *C3orf20* gene in the testis. (**b**) Normalized mRNA expression level of the *AC090952.5* gene in the testis. (**c**) Normalized mRNA expression level of the *WNT7A* gene in the spinal cord.
